# Supplementary material for: Multivariable regulation of gene expression plasticity in metazoans
Source: Open Biol. 2019 Dec 4;9(12):190150. doi: 10.1098/rsob.190150 (PMC6936262; doi:10.1098/rsob.190150)
Supplement: Supplementary figures [file rsob190150supp1.pdf]

Supplementary Data

1. Supplementary figures

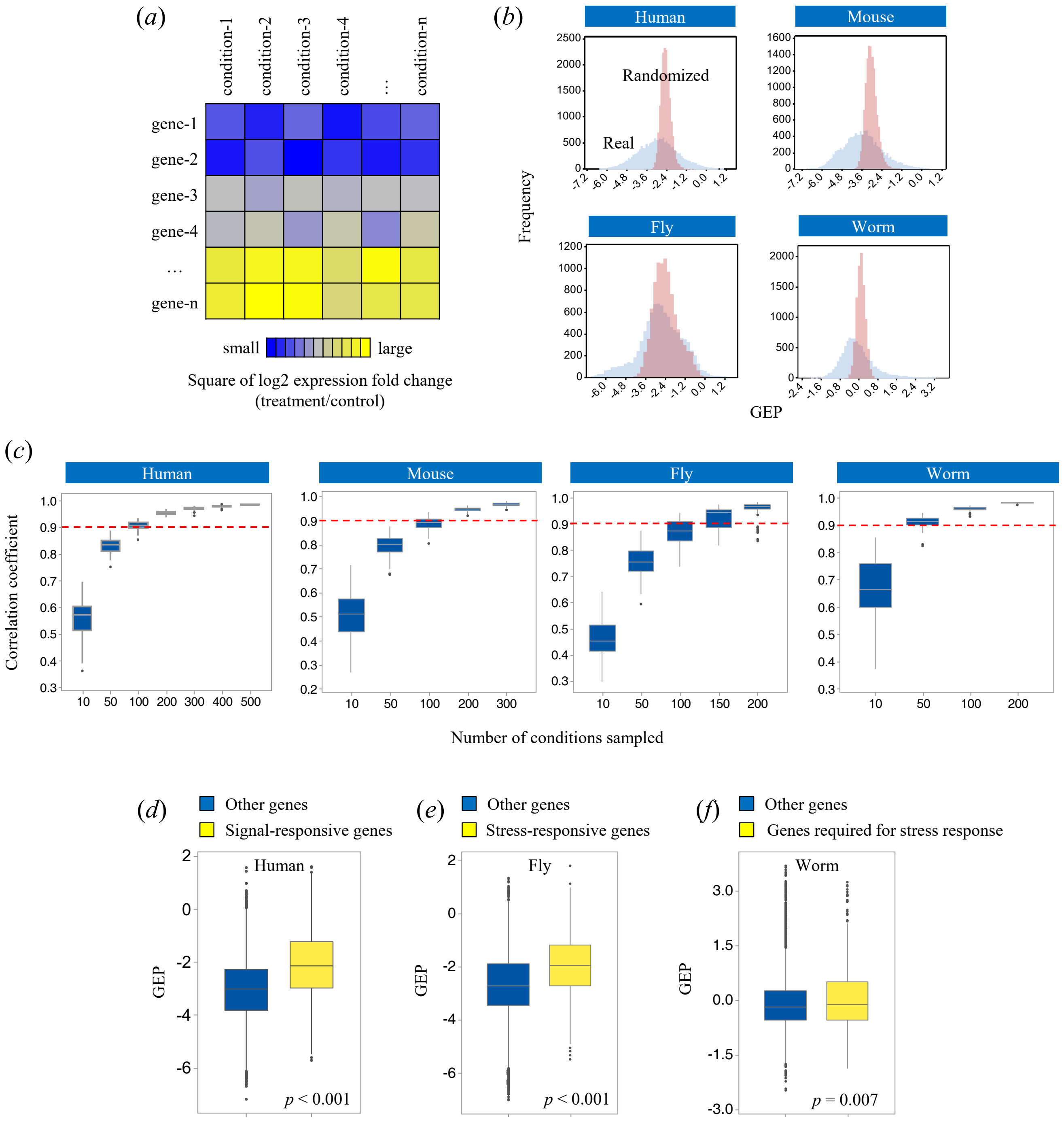

Figure S1. Quality control of gene expression plasticity. (*a*) Graphic showing the strategy for quantifying GEP from changes of gene expression measured across diverse conditions. (*b*) Distribution of expected and observed GEP values in four species. Light blue shows the observed distribution of GEP values and light red shows that of expected distribution when expression changes were randomized across genes in all conditions. (*c*) Sufficiency of condition number for GEP quantification. The influence of condition numbers on GEP quantification was simulated by comparing the Pearson correlation coefficients of GEP values calculated using all conditions and those using only a subset of all conditions in all species. Each sampling was repeated 100 times. Red dashed line indicates a correlation coefficient of 0.9. (*d*) Human signal-responsive genes (n = 2,221) [1] exhibit significantly higher GEP than other genes. (*e-f*) Genes implicated in stress response show significantly higher GEP. Fly stress response genes (*e*, n = 891) were identified previously [2]; worm genes (*f*, n = 505) required for stress response were those whose inactivation (by RNAi or mutation) caused hypersensitivity to stress conditions obtained from WormBase [3].

(*a*)

Organism	Gene number	Spearman's Rank Correlation
Human-Mouse	10,260	ρ = 0.46 ***
Human-Fly	2,102	ρ = 0.15 ***
Human-Worm	904	ρ = 0.12 ***
Mouse-Fly	1,569	ρ = 0.18 ***
Mouse-Worm	687	ρ = 0.17 ***
Fly-Worm	1,075	ρ = 0.14 ***

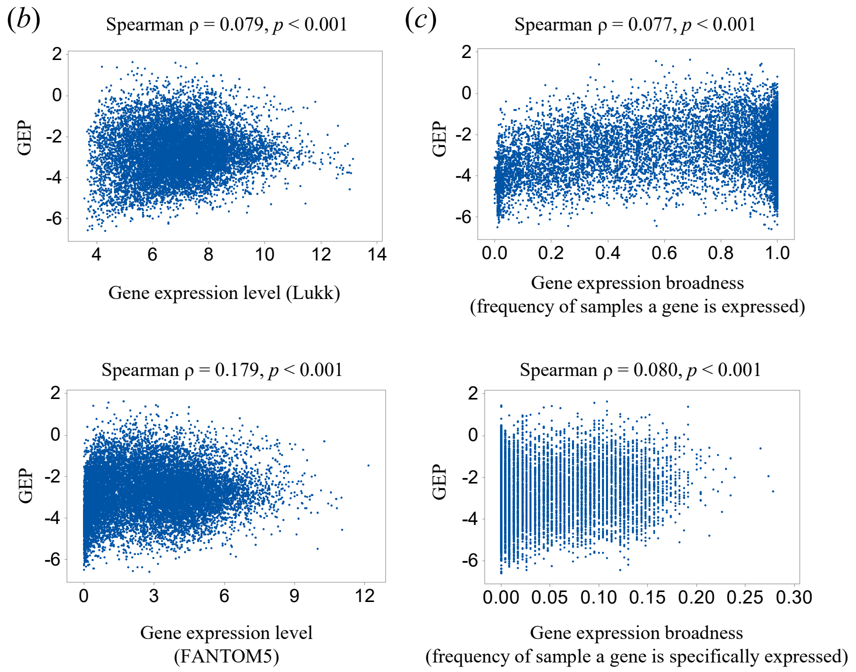

Figure S2. Gene expression plasticity is conserved across species and measure a distinct feature of gene expression. (*a*) GEP of orthologous genes is conserved in four metazoan species. Pairwise correlation coefficients were calculated for GEP of one-to-one orthologous genes in four metazoan species [4]. Figure shows the overall Spearman's rank correlation coefficient between species. *** denotes $p < 0.001$. (*b*) GEP is poorly correlated with gene expression level. Scatterplot compares GEP and expression level measured using two different datasets. (*c*) GEP is poorly correlated with gene expression broadness measured using two different methods.

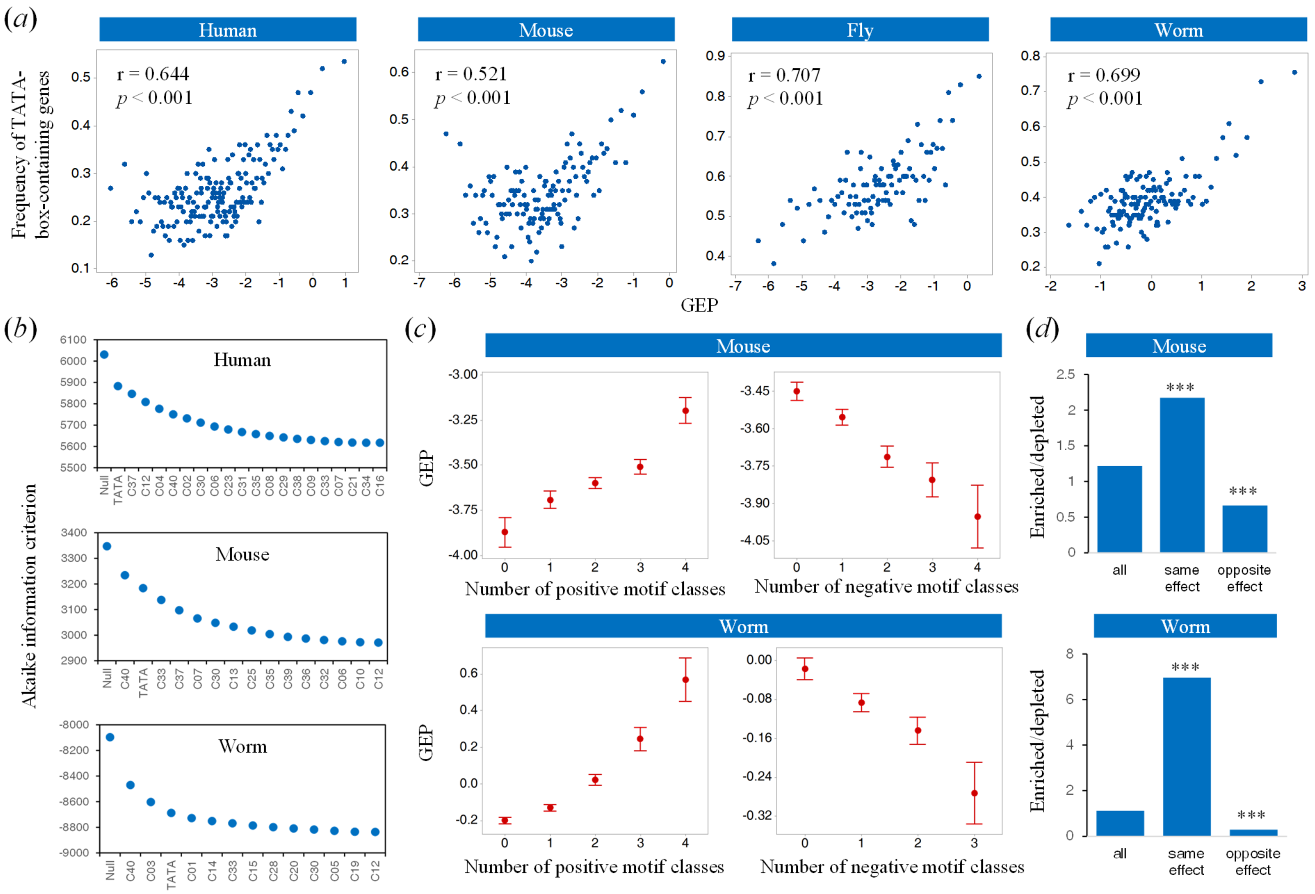

(e)

Motif 1	Motif 2	Enhancement	Dominance (motif 1 masks 2)	Dominance (motif 2 masks 1)	Additive
Promoting	Promoting	$GEP_{obs}>GEP_{exp}$<br>$GEP_{obs}>GEP_{M1}$<br>$GEP_{obs}>GEP_{M2}$	NA	NA	$GEP_{obs}=GEP_{exp}$
Repressive	Repressive	$GEP_{obs}<GEP_{exp}$<br>$GEP_{obs}<GEP_{M1}$<br>$GEP_{obs}<GEP_{M2}$	NA	NA	$GEP_{obs}=GEP_{exp}$
Promoting	Repressive	NA	$GEP_{obs}>GEP_{exp}$<br>$GEP_{obs}=GEP_{M1}$<br>$GEP_{obs}>GEP_{M2}$	$GEP_{obs}<GEP_{exp}$<br>$GEP_{obs}<GEP_{M1}$<br>$GEP_{obs}=GEP_{M2}$	$GEP_{obs}=GEP_{exp}$
Repressive	Promoting	NA	$GEP_{obs}<GEP_{exp}$<br>$GEP_{obs}=GEP_{M1}$<br>$GEP_{obs}<GEP_{M2}$	$GEP_{obs}>GEP_{exp}$<br>$GEP_{obs}>GEP_{M1}$<br>$GEP_{obs}=GEP_{M2}$	$GEP_{obs}=GEP_{exp}$

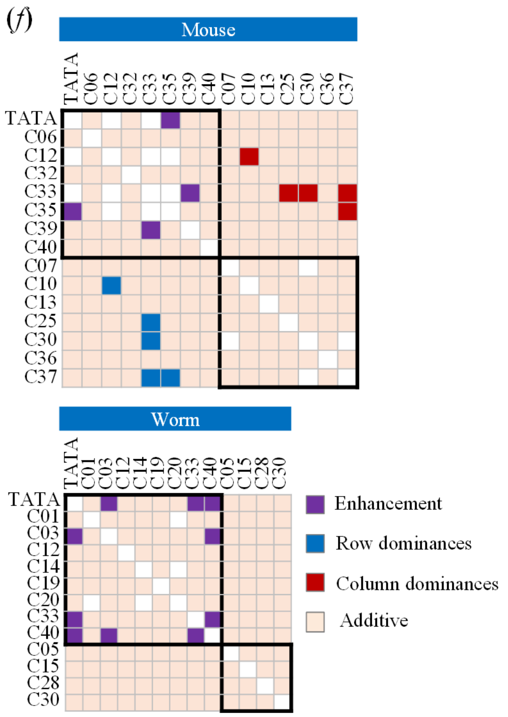

Figure S3 *Cis*-regulation of gene expression plasticity. (*a*) Promoter TATA box is associated with high GEP in four metazoan species. Genes were ordered by GEP and the frequency of TATA box-containing genes was calculated for each bin of 100 genes. (*b*) Contributions of DNA motif classes to GEP. Figure shows the reduction of Akaike information criterion (AIC) score following inclusion of all selected motif classes in the predictive model for each species. Motif selection was performed using a stepwise AIC procedure to identify the contribution of each motif class after considering the influence of other motifs. (*c*) Cumulative effect of motifs. Changes in GEP as a function of more GEP-promoting (left) or GEP-repressing (right) motif classes in promoters of mouse and worm genes. (*d*) Optimization of motif co-occurrence. Bar graph gives the ratios of enriched to depleted co-occurrence of two DNA motif classes for all pairwise motif pairs (all) and motif pairs that influence GEP in the same or opposite directions. Enriched and depleted motif pairs are defined as those with observed co-occurrence significantly higher or lower than expected (Fisher's exact test, $p < 0.05$), respectively. *** denotes ratio significantly different from that of all motif pairs (Fisher's exact test, $p < 0.001$). (*e*) Rules used to identify interactions between pairwise motifs. Details described in Methods section. (*f*) Combinatorial effects of motif classes on GEP in mouse and worm. Black boxes highlight motif classes that affect GEP in the same direction.

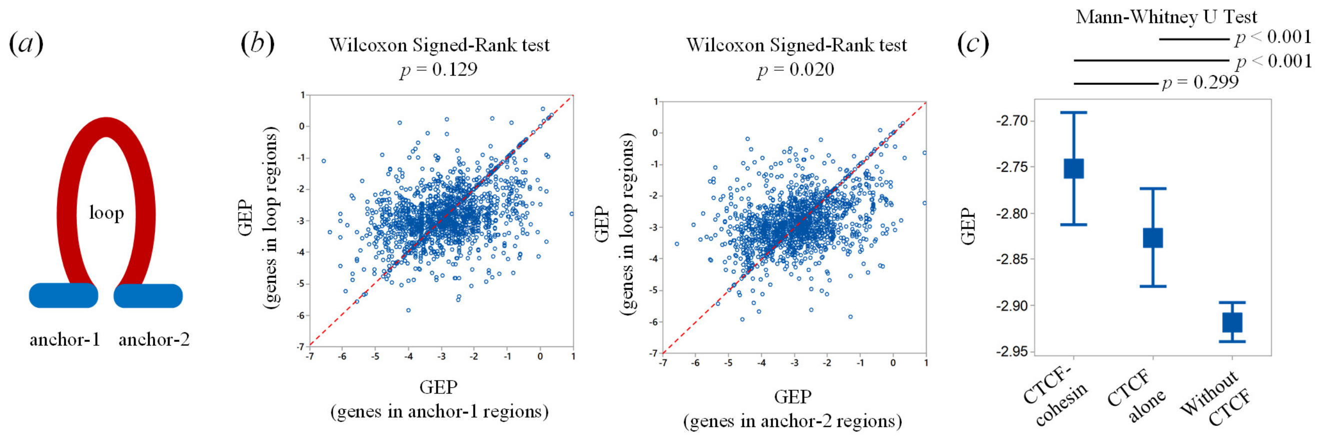

Figure S4. CTCF-mediated chromatin looping is not associated with expression plasticity. (*a*) Schematic of chromatin loop structure. (*b*) Scatterplots compare GEP between genes with promoters located within anchors (X-axis, n = 2139 for anchor-1 and n = 2,084 for anchor-2) and those with promoters located in loop regions (Y-axis). If promoters of multiple genes are present in an anchor or loop region, their GEP was averaged. Diagonal line indicates equal GEP levels. (*c*) Comparison of GEP between genes whose promoter is bound by CTCF-cohesin (associated with chromatin loop, n = 1,469), bound by CTCF alone (n = 1,685), and not bound by CTCF (n = 12,545). Data is represented as mean ± 95% confidence interval.

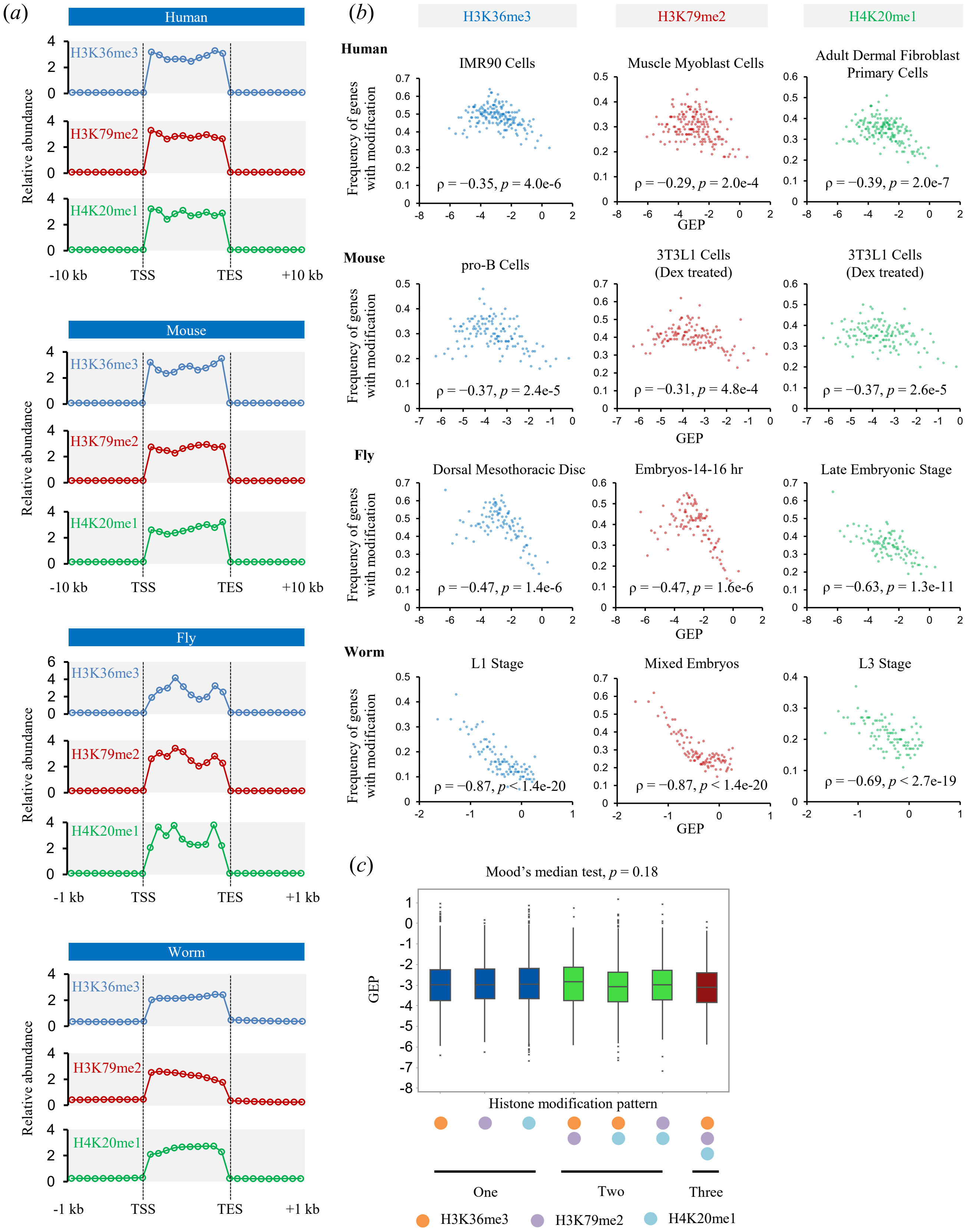
(a)
Human
H3K36me3
H3K79me2
H4K20me1
Relative abundance
-10 kb
TSS
TES
+10 kb
Mouse
Fly
-1 kb
+1 kb
Worm
(b)
H3K36me3
H3K79me2
H4K20me1
Human
IMR90 Cells
Muscle Myoblast Cells
Adult Dermal Fibroblast Primary Cells
Frequency of genes with modification
ρ = −0.35, p = 4.0e-6
ρ = −0.29, p = 2.0e-4
ρ = −0.39, p = 2.0e-7
GEP
Mouse
pro-B Cells
3T3L1 Cells (Dex treated)
3T3L1 Cells (Dex treated)
ρ = −0.37, p = 2.4e-5
ρ = −0.31, p = 4.8e-4
ρ = −0.37, p = 2.6e-5
Fly
Dorsal Mesothoracic Disc
Embryos-14-16 hr
Late Embryonic Stage
ρ = −0.47, p = 1.4e-6
ρ = −0.47, p = 1.6e-6
ρ = −0.63, p = 1.3e-11
Worm
L1 Stage
Mixed Embryos
L3 Stage
ρ = −0.87, p < 1.4e-20
ρ = −0.87, p < 1.4e-20
ρ = −0.69, p < 2.7e-19
(c)
Mood's median test, p = 0.18
GEP
Histone modification pattern
One
Two
Three
H3K36me3
H3K79me2
H4K20me1

Figure S5. Influence of gene body histone modifications on gene expression plasticity. (*a*) Enrichment of H3K36me3, H3K79me2, and H4K20me1 in gene body. Figure shows the relative abundance of H3K36me3 (blue), H3K79me2 (red), and H4K20me1 (green) modifications in genomic regions upstream of transcription state sites, the gene body and downstream of transcription termination sites in four species. For human and mouse genomes, 10-kb regions on each side of the gene body was analysed. Only 1-kb regions on both side of the gene body were analysed for fly and worm genomes due to short intergenic regions. The upstream, gene body and downstream regions were divided into 10 bins and the modification abundance was measured as number of peaks per kb and averaged across all genes. The abundance value in each bin was further normalized to the mean value of all bins to represent the relative abundance. Each plot shows the average distribution profile of all biological samples for each modification in each species. (*b*) Gene body H3K36me3, H3K79me2, and H4K20me1 are associated with lower GEP. Genes were ranked by expression plasticity and the average frequency of genes enriched for H3K36me3 (blue), H3K79me2 (red), and H4K20me1 (green) modifications in the gene body was calculated for each bin (100 genes). One representative sample for each modification are shown for each species. (*c*) Comparison of GEP of genes with different combinations of the three histone modifications in the gene body regions.

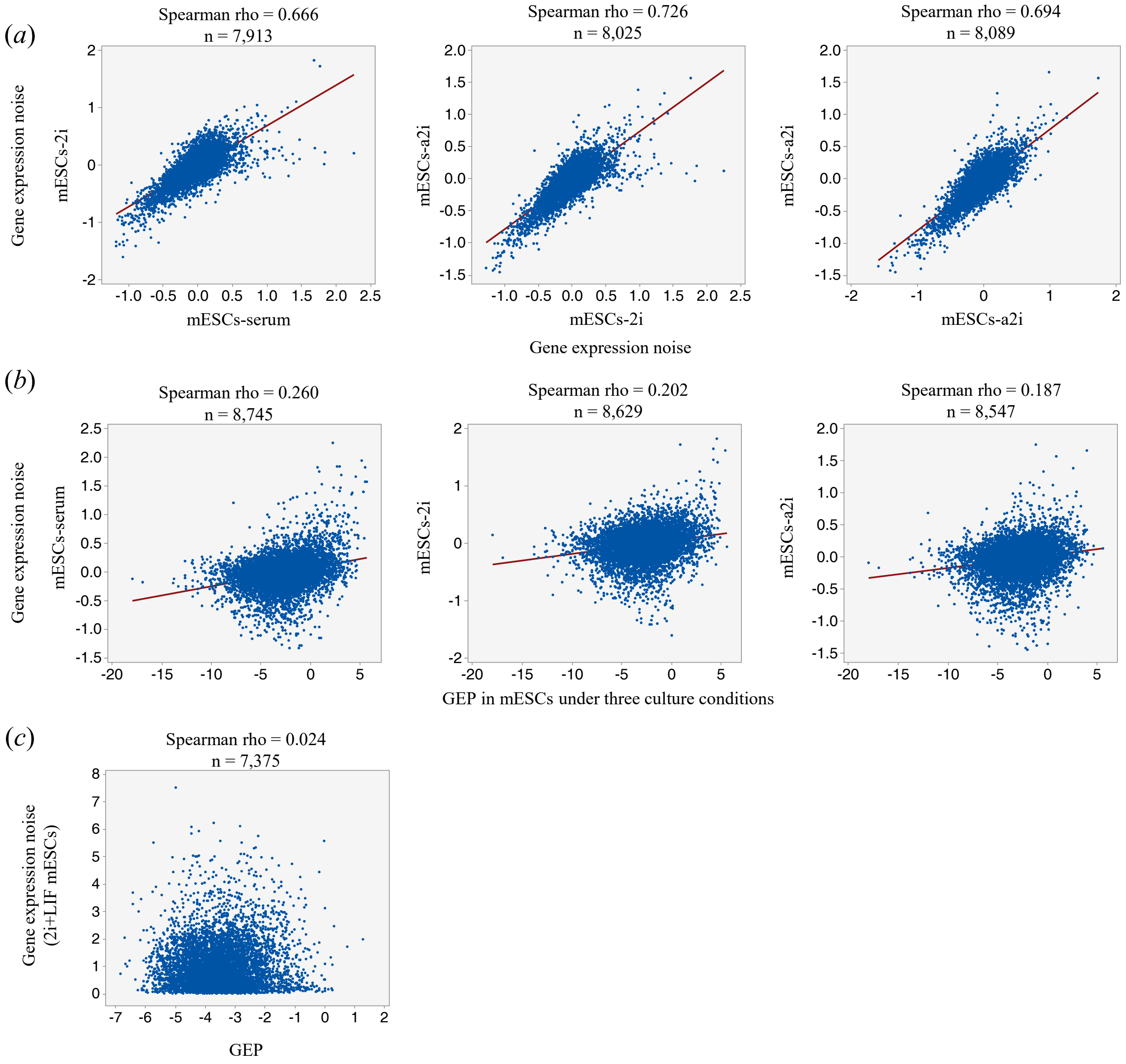

Figure S6. Decoupling of gene expression plasticity and noise. (*a*) Correlation of gene expression noise under different culture conditions. Scatterplots show pairwise correlations of gene expression noise in mouse embryonic stem cells (mESCs) cultured under three different conditions (serum, 2i, and a2i). Gene expression noise was determined by the original study [5]. (*b*) Correlation between GEP and gene expression noise in mESCs. Scatterplots show the correlation between GEP in mESCs and expression noise in three culture conditions. GEP in mESCs was measured using the same method as in Figure 1 by determining the magnitudes of gene expression fold changes between the culture conditions. (*c*) Scatter plot shows correlation between GEP and gene expression noise in which the same types of histone modifications are identified to restrict GEP and expression noise respectively. As in the previous study [6], coefficients of variation of gene expression levels in 94 single mESCs cultured under 2i+LIF conditions [7] were used to quantify expression noise.

(*a*)

GRO-seq Dataset	GEP of genes without Pol II pausing	GEP of genes with Pol II pausing	Mann-Whitney U test *p* value
GSM2706105_MCF7	−2.8387<br>n = 9,375	−2.8532<br>n = 4,689	0.0436
GSM2706107_MCF7	−2.8355<br>n = 9,353	−2.8481<br>n = 4,608	0.1595
GSE62046_HeLa	−2.8318<br>n = 5,658	−2.8145<br>n = 7,381	0.4723
GSM2551016_HEK293T	−2.8503<br>n = 8,703	−2.8144<br>n = 4,601	0.0479

(*b*)

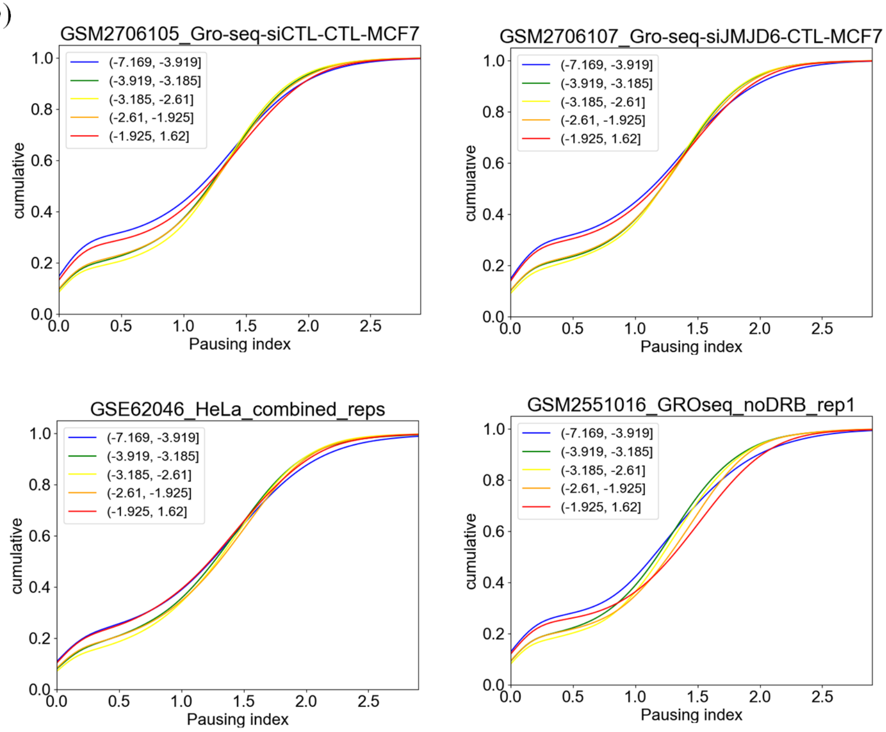

Figure S7. RNA Pol II pausing is not associated with GEP. (*a*) Genes with or without Pol II pausing exhibit similar GEP values. RNA Pol II pausing was determined from four Global Run-on Sequencing (GRO-seq) datasets [8-10] using the groHMM algorithm [11]. Nascent mRNA read densities were compared between promoter-proximal regions (−50 to +300 relative to the TSS) and in gene body regions (from +300 relative to the TSS to +3000 from the gene end) and genes were identified as having Pol II pausing if disproportionately high (Fisher's exact test, $p < 0.001$) read density was present in the promoter-proximal region. Only actively transcribed genes were analysed, as determined by significantly enriched ($p < 0.01$) read density in the gene body compared to background (bottom 1% read density of all genes), as done in a previous study [12]. (*b*) Comparison of Pol II pausing index between genes with different GEP. Genes were divided into five bins according to GEP levels and the cumulative distribution of Pol II pausing index (log2 transformed) plotted. The pausing index was calculated as the ratio of read density in promoter-proximal regions to that of gene body regions for all actively transcribed genes.

2. Supplementary table legends

Table S1. GEP of four metazoan species. Sheets 1 to 4. Gene expression plasticity of human, mouse, fly, and worm genes.

Table S2. GEP of benchmark genes. Sheet 1- 3. GEP of human signal-responsive genes, fly stress-responsive genes, worm genes required for stress response.

Table S3. Comparison of GEP measured based on challenging and normal conditions. GEP was normalized by rank percentile for comparison. 1 denotes signal-responsive genes.

Table S4. Comparison of GEP between orthologs. Sheet 1-4. GEP of human, mouse, fly and worm genes and corresponding one-to-one orthologs in other species.

Table S5. Comparison of GEP to gene expression level and broadness. Sheet 1. GEP and average gene expression levels across diverse samples for human genes. Sheet 2. GEP and gene expression broadness across diverse samples for human genes.

Table S6. GEP of genes with specific function. Sheet 1-3. GEP of homeobox genes, hormone and receptor genes and innate-immunity genes. 1 denotes a gene belongs to a specific category.

Table S7. Cross-species comparison of GEP between genes with similar function. Sheet 1-6. Comparison of average GEP of all genes with an identical Gene Ontology term between all pairwise species.

Table S8. GEP and disease susceptibility. Table shows GEP of genes associated with diseases and related to cancer.

Table S9. Core promoter motifs associated with GEP. Sheet 1. Identified DNA motifs, effects on GEP, motif classes, and associated factors in four species. Sheets 2-4. Presence of motif classes in human, mouse, and worm genes.

Table S10. Binding of regulatory proteins at the promoter is associated with GEP. Sheet 1. Effects of all analysed regulatory proteins on expression plasticity in all samples. Sheet 2. Summary of results.

Table S11. Data sources for histone modifications. Table shows data sources for H3K36me3, H3K79me2, and H4K20m1 modifications for all species.

Table S12. H3K36me3, H3K79me2 and H4K20me1 are associated with low GEP. Sheet 1-4. Comparison of GEP between genes with or without a histone modification in the gene body in four species.

Table S13. No combinatorial effects of H3K36me3, H3K79me2 and H4K20me1 on GEP. Table compares GEP of genes with different combinations of H3K36me3/H3K79me2/H4K20me1 modifications in the gene body region.

3. Supplementary references

1 Kandasamy, K., Mohan, S. S., Raju, R., Keerthikumar, S., Kumar, G. S., Venugopal, A. K., Telikicherla, D., Navarro, J. D., Mathivanan, S., Pecquet, C.*, et al.* 2010 NetPath: a public resource of curated signal transduction pathways. *Genome Biol*. **11**, R3. (10.1186/gb-2010-11-1-r3)

2 Girardot, F., Monnier, V., Tricoire, H. 2004 Genome wide analysis of common and specific stress responses in adult drosophila melanogaster. *BMC Genomics*. **5**, 74. (10.1186/1471-2164-5-74)

3 Howe, K. L., Bolt, B. J., Cain, S., Chan, J., Chen, W. J., Davis, P., Done, J., Down, T., Gao, S., Grove, C.*, et al.* 2016 WormBase 2016: expanding to enable helminth genomic research. *Nucleic Acids Res*. **44**, D774-780. (10.1093/nar/gkv1217)

4 Herrero, J., Muffato, M., Beal, K., Fitzgerald, S., Gordon, L., Pignatelli, M., Vilella, A. J., Searle, S. M., Amode, R., Brent, S.*, et al.* 2016 Ensembl comparative genomics resources. *Database : the journal of biological databases and curation*. **2016**, (10.1093/database/baw053)

5 Kolodziejczyk, A. A., Kim, J. K., Tsang, J. C., Ilicic, T., Henriksson, J., Natarajan, K. N., Tuck, A. C., Gao, X., Buhler, M., Liu, P.*, et al.* 2015 Single Cell RNA-Sequencing of Pluripotent States Unlocks Modular Transcriptional Variation. *Cell Stem Cell*. **17**, 471-485. (10.1016/j.stem.2015.09.011)

6 Wu, S., Li, K., Li, Y., Zhao, T., Li, T., Yang, Y. F., Qian, W. 2017 Independent regulation of gene expression level and noise by histone modifications. *PLoS Comput Biol*. **13**, e1005585. (10.1371/journal.pcbi.1005585)

7 Kumar, R. M., Cahan, P., Shalek, A. K., Satija, R., DaleyKeyser, A., Li, H., Zhang, J., Pardee, K., Gennert, D., Trombetta, J. J.*, et al.* 2014 Deconstructing transcriptional heterogeneity in pluripotent stem cells. *Nature*. **516**, 56-61. (10.1038/nature13920)

8 Gao, W. W., Xiao, R. Q., Zhang, W. J., Hu, Y. R., Peng, B. L., Li, W. J., He, Y. H., Shen, H. F., Ding, J. C., Huang, Q. X.*, et al.* 2018 JMJD6 Licenses ERalpha-Dependent Enhancer and Coding Gene Activation by Modulating the Recruitment of the CARM1/MED12 Co-activator Complex. *Mol Cell*. **70**, 340-357 e348. (10.1016/j.molcel.2018.03.006)

9 Andersson, R., Refsing Andersen, P., Valen, E., Core, L. J., Bornholdt, J., Boyd, M., Heick Jensen, T., Sandelin, A. 2014 Nuclear stability and transcriptional directionality separate functionally distinct RNA species. *Nat Commun*. **5**, 5336. (10.1038/ncomms6336)

10 Chen, L., Chen, J. Y., Zhang, X., Gu, Y., Xiao, R., Shao, C., Tang, P., Qian, H., Luo, D., Li, H.*, et al.* 2017 R-ChIP Using Inactive RNase H Reveals Dynamic Coupling of R-loops with Transcriptional Pausing at Gene Promoters. *Mol Cell*. **68**, 745-757 e745. (10.1016/j.molcel.2017.10.008)

11 Chae, M., Danko, C. G., Kraus, W. L. 2015 groHMM: a computational tool for identifying unannotated and cell type-specific transcription units from global run-on sequencing data. *BMC Bioinformatics*. **16**, 222. (10.1186/s12859-015-0656-3)

12 Core, L. J., Waterfall, J. J., Gilchrist, D. A., Fargo, D. C., Kwak, H., Adelman, K., Lis, J. T. 2012 Defining the status of RNA polymerase at promoters. *Cell reports*. **2**, 1025-1035. (10.1016/j.celrep.2012.08.034)
